# Micro-scale environment and mental health in later life: Results from the Cognitive Function and Ageing Study II (CFAS II)

**DOI:** 10.1016/j.jad.2017.05.001

**Published:** 2017-08-15

**Authors:** Yu-Tzu Wu, A. Matthew Prina, Andy Jones, Linda E. Barnes, Fiona E. Matthews, Carol Brayne, MRC CFAS

**Affiliations:** a Department of Public Health and Primary Care, Institute of Public Health, Forvie Site, University of Cambridge, School of Clinical Medicine, Cambridge Biomedical Campus, Cambridge CB2 0SR, United Kingdom; b King’s College London, Institute of Psychiatry, Psychology and Neuroscience, Centre for Global Mental Health, Health Service and Population Research Department, De Crespigny Park, Denmark Hill, London SE5 8AF, United Kingdom; c Norwich Medical School, University of East Anglia, Norwich, Norfolk NR4 7TJ, United Kingdom; d MRC Biostatistics Unit, Institute of Public Health, University of Cambridge, Cambridge CB2 0SR, United Kingdom; e Institute of Health and Society, Newcastle University, Newcastle upon Tyne NE4 5PL, United Kingdom

**Keywords:** Environment, Neighbourhood, Older age, Depression, Anxiety, Cognitive disorder

## Abstract

**Background:**

Poor micro-scale environmental features, such as graffiti and broken windows, have been associated with crime and signs of social disorder with a potential impact on mental health. The aim of this study is to investigate the association between micro-scale environment and mental health problems in later life, including cognitive (cognitive impairment and dementia) and common mental disorders (depressive and anxiety symptoms).

**Methods:**

The method of visual image audits was used to collect micro-scale environmental data for 3590 participants in the Cognitive Function and Ageing Study II, a population-based multicentre cohort of people aged 65 or above in England. Multilevel logistic regression was used to examine the associations between the quality of micro-scale environment and mental health problems taking into account urban/rural difference.

**Results:**

Poor quality of micro-scale environment was associated with nearly 20% increased odds of depressive (OR: 1.19; 95% CI: 0.99, 1.44) and anxiety symptoms (OR: 1.17; 95% CI: 0.99, 1.38) while the direction of association for cognitive disorders differed across urban and rural settings. Although higher odds of cognitive disorders were found in rural settings, living in a poor quality environment was associated with nearly twice higher odds of cognitive impairment (OR: 1.88; 95% CI: 1.18, 2.97) in urban conurbations but 20% lower odds in rural areas (OR: 0.80; 95% CI: 0.57, 1.11).

**Limitations:**

The causal direction could not be fully determined due to the cross-sectional nature of the data. The visual nature of the environmental assessment tool means it likely does not fully capture features related to the availability of local support services, or opportunities for social participation and interaction.

**Conclusions:**

The quality of micro-scale environment appears to be important to mental health in older people. Interventions may incorporate the environmental aspect to reduce cognitive and common mental disorders.

## Highlights

• Poor micro-scale features were related to depression and anxiety in later life.
• The association for cognitive disorders differed across urban and rural settings.
• Improvement of micro-scale environment may support mental health in older people.

## 1 Introduction

There has been a growing interest in identifying environmental factors related to mental disorders (Mair et al., 2008). Physical characteristics at the micro-scale level, such as poor pavement condition, graffiti, vandalism and litter, have been reported to be related to social disorder and lack of informal control in local areas (Kelling and Wilson, 1982, Blair. et al., 2014) and might have a potential impact on stress and insecurity as well as increasing the risk of mental illnesses (Blair. et al., 2014, Julien et al., 2012). Although these detailed features are advocated as providing additional information on the living environment, they cannot be captured from the existing collection of small area statistics. To collect these micro-scale features, traditional physical audit has involved assessors visiting local areas and rating the environment and micro-scale features at particular times. This approach can be time-consuming and subject to several unpredictable factors, such as inclement weather or poor transport links, which may influence the rate of progress and safety of assessors (Charreire et al., 2014). A small number of existing studies have investigated the associations between mental disorders and some micro-scale features but these studies have been small with limited statistical power to measure substantial effect sizes (Weich et al., 2002, Thomas et al., 2007, Araya et al., 2007, Galea et al., 2005).

In addition to common mental disorders, recent studies have also identified environmental factors that may be related to cognitive disorders in later life. Evidence from epidemiological research supports the presence of urban/rural differences in dementia prevalence and cognitive function (Cassarino et al., 2016, Nunes et al., 2010, Russ et al., 2012). Area deprivation and some physical and social environmental factors such as features of land use, access to local services and resources, have also been related to cognitive function in older people (Clarke et al., 2012; Lang et al., 2008; Watts et al., 2015; Wu et al., 2015a, Wu et al., 2015b). However, these studies have mainly focused on small area or neighbourhood level characteristics and few have investigated the potential impact of micro-scale features. This is limiting as poor quality micro-scale environments might be related to stress or an overload of sensory stimulation, with a negative impact on cognitive function (Cassarino and Setti, 2015, Marchant and Howard, 2015).

In the recent five years, some studies have used visual streetscape images, including Google Street View, Bing Maps or omnidirectional imagery, to observe these micro-scale features and show good inter-method reliability between visual image audits and physical audits (Charreire et al., 2014). In order to assess micro-scale level features in a large population, in an earlier study (Wu et al., 2014) we have previously developed and validated the method of visual image audits based on the Residential Environmental Assessment Tool (REAT) (Dunstan et al., 2005), a UK-based instrument designed for assessing specific micro-scale features within a postcode unit. This method has been applied to collect environmental data for a sub-sample of the Cognitive Function and Ageing Study II (CFAS II), a population-based cohort of people aged 65 or over across rural and urban areas in England. The aim of this study is to examine the relationship between the quality of micro-scale environment (the REAT scores) and cognitive and common mental disorders, two important aspects of mental health in later life, taking rural and urban contexts into account.

## 2 Methods

### 2.1 Study population

This study was based on the Cognitive Function and Ageing Study II (CFAS II), a longitudinal population-based cohort of people aged 65 or above across three areas in England (Cambridgeshire, Newcastle and Nottingham). Details of CFAS II have been described elsewhere (Matthews et al., 2013). In brief, the study population was sampled from primary care registrations. Each study area included 2500 individuals with equal sample sizes of those aged 65–74 years and ≥75 years. Eligible participants were sent an introductory letter by their General Practitioner and this was followed by a visit from a study interviewer. Informed written consent was obtained from those who agreed to take part. Interviewers were trained to deliver standardised computerised questionnaires in participants’ residences and collected information on socio-demographics, lifestyle, health status, cognitive function and psychiatric symptoms. Among the 14,242 people approached, the baseline interview included 7796 people living in community settings and institutions between 2008 and 2011.

The analysis here was based on a sub-sample of 3590 (47.8%) community-based CFAS II participants. A random sample of postcodes in Cambridgeshire and Nottingham was selected to collect environmental data, which was linked to the baseline interview data. The choice of these two centres was based on urban/rural difference and geographical variation in health status and social disadvantage (Matthews et al., 2013). CFAS II was approved by relevant local research ethics committees and obtained informed consent from participants. This secondary data analysis does not require new ethical approval.

### 2.2 Individual level factors and mental health problems

Socio-demographic factors were obtained from the CFAS II baseline interview, including age, gender and education (less than 9 years, 10–11 years, 12 years and above). Self-reported information on co-morbidity was used to measure the number of chronic conditions (hypertension, diabetes, stroke, heart attack, angina, low blood pressure/dizzy on standing, hearing and vision impairment) and divided into three levels (none, one, two or more).

Cognitive and common mental disorders were assessed using a structured interview of psychiatric symptoms and standardised cognitive tests. Cognitive impairment was defined here by Mini-Mental State Examination (MMSE) score 25 or below (Folstein et al., 1975). Dementia, depressive and anxiety symptoms were identified by Geriatric Mental Status (GMS) and the algorithm of the Automatic Geriatric Examination for Computer Assisting Taxonomy (GMS-AGECAT) (Copeland et al., 1986). People with organicity level three or above were considered to be dementia cases. Depressive and anxiety symptoms were defined as an AGECAT depression level of one or above including all subthreshold and clinical cases.

### 2.3 Residential Environmental Assessment Tool (REAT)

Environmental features at the micro-scale level were measured using the Residential Environmental Assessment Tool (REAT), a validated and observational instrument designed for measuring the quality of living environment within a given UK postcode, which includes approximately 18 households on average (Dunstan et al., 2005, Office for National Statistics, 2013a). The REAT contains property and street level assessments by examining 28 items in four domains: physical incivilities (features of social disorder, such as graffiti, broken windows, litter on the street); territorial functioning (maintenance and management of private areas, such as decorative features in gardens); defensible space (designs encouraging the control of local areas, such as walls and fences separating public and private areas) and natural elements (aesthetic and outlook of streets, such as trees and green space). A higher REAT score indicates a worse quality of living environment in the postcode.

In this study, the REAT assessment was conducted through visual image audits, which used Google Street View to virtually ‘walk through’ streets and assess the quality of living environment in local areas. The reliability of physical and visual image audits has been validated in our earlier study (Wu et al., 2014). Visual image audits show acceptable reliability in the total REAT score but nevertheless individual items and domain scores need to be treated with caution, particularly multiple comparisons. Thus, the analysis here only focused on total REAT score. The total REAT score was divided into two groups (high and low) based on the median score (high REAT score group: 2.0–14.0; low REAT score group: 14.5–41.0).

### 2.4 Urban and rural classification

Postcodes of CFAS II participants were mapped onto Lower Super Output Areas (LSOA), small geographical units in the UK with an average of 1500 people per area, and linked to corresponding rural/urban categories in the 2011 Rural/Urban Classification for Small Areas Geographies (UK Government, Department for Environment, Food and Rural Affairs, 2013a). The classification is based on the characteristics of physical settlement and population sparsity (UK Government, Department for Environment, Food and Rural Affairs, 2013a). There were three urban categories: Major Conurbation (mean population density (PD): 35.5 people per hectare), Minor Conurbation (PD: 22.6), City and Town (PD: 16.5); and two rural categories: Town and Fringe (PD: 5.9), Village and Dispersed (PD: 0.5) (Office for National Statistics, 2013b). In order to increase the statistical power of the analyses, these urban and rural categories were combined into three types: Urban Conurbation (Major and Minor Conurbation), Urban City and Town and Rural areas (Town and Fringe, Village and Dispersed) based on the similarity of their environmental features.

### 2.5 Statistical analysis

A small number of postcodes had missing REAT data due to unavailable or unclear streetscape images. Since the distributions of individual level factors and urban rural categories were different across those with complete and missing REAT data, inverse probability weighting was used to adjust for missing data. The weights were calculated by age, gender, education, cognitive impairment, depressive and anxiety symptoms and rural/urban category.

The association between mental health problems and environmental measurements was investigated by multilevel logistic regression. The analysis first examined the overall association between cognitive disorders, common mental disorders and the REAT groups adjusting for individual level factors (age, gender, education and chronic conditions), and missing data. To further take into account rural/urban difference in this sub-sample, interaction terms between the binary REAT groups and rural/urban categories were added to regression modelling to investigate whether the association between micro-scale environmental features and mental health problems differed according to urban/rural context. A likelihood ratio test was conducted to investigate the statistical significance of the interaction terms.

A sensitivity analysis was conducted to further test for potential bias due to missing data. Based on our investigation, postcodes with missing data were more likely to comprise a poor quality residential environment (Supporting information S1). The 426 people (11.9%) with missing data were allocated to the high REAT group to investigate whether the estimates changed considerably after including the missing REAT data.

## 3 Results

The REAT assessment was applied to 3590 CFAS II participants and 3164 (88%) had complete data (Table 1). Among the 3590 people aged 65 or above, over 25% had an MMSE score less than 25% and 4.4% had dementia. For common mental disorders, the prevalence was 23.7% for depressive symptoms and 33.0% for anxiety symptoms. The median score for those with complete REAT data was 14.0 (interquartile range (IQR): 8.0) with a range between 2.0 and 41.0. Median scores were higher in urban conurbations (16.0; IQR: 8.0) than urban city and town (13.0; IQR: 7.0) and rural areas (9.0; IQR: 7.5). The distributions of socio-demographic factors, mental health problems and urban/rural category were different between those with complete and missing REAT data. The missing data group (N=426) were older, urban residents and had higher prevalence of cognitive impairment, depressive and anxiety symptoms compared to those with complete data.

**Table 1:** Distributions of individual level factors and mental health problems in the analysis (N (%)).

	Complete REAT data	Missing REAT data	Total
N	3164	426	3590
Age group			
65–69	886 (28.0)	90 (21.1)	976 (27.2)
70–74	736 (23.3)	94 (22.1)	830 (23.1)
75–79	672 (21.2)	79 (18.5)	751 (20.9)
80–84	507 (16.0)	86 (20.2)	593 (16.5)
85+	363 (11.5)	77 (18.1)	440 (12.6)
Gender			
Women	1646 (52.0)	247 (58.0)	1893 (52.7)
Men	1518 (48.0)	179 (42.0)	1697 (47.3)
Education			
12 years and above	798 (25.4)	96 (22.9)	894 (25.1)
10–11 years	1590 (50.6)	191 (45.5)	1781 (50.0)
9 year and below	753 (24.0)	133 (31.7)	886 (24.9)
Number of chronic illness			
None	804 (25.4)	101 (23.7)	905 (25.2)
One	1044 (33.0)	121 (28.4)	1165 (32.5)
Two and more	1316 (41.6)	204 (47.9)	1520 (42.3)
Cognitive impairment (MMSE<25)	770 (24.5)	124 (29.5)	894 (25.1)
Dementia	142 (4.5)	16 (3.8)	158 (4.4)
Depressive symptoms	718 (22.8)	127 (30.0)	845 (23.7)
Anxiety symptoms	1014 (32.3)	161 (38.1)	1175 (33.0)
Urban/rural category			
Urban conurbation	871 (27.5)	173 (40.6)	1044 (29.1)
Urban city and town	931 (29.4)	111 (26.1)	1042 (29.0)
Rural areas	1362 (43.1)	142 (33.3)	1504 (41.9)

**Fig. 1:**
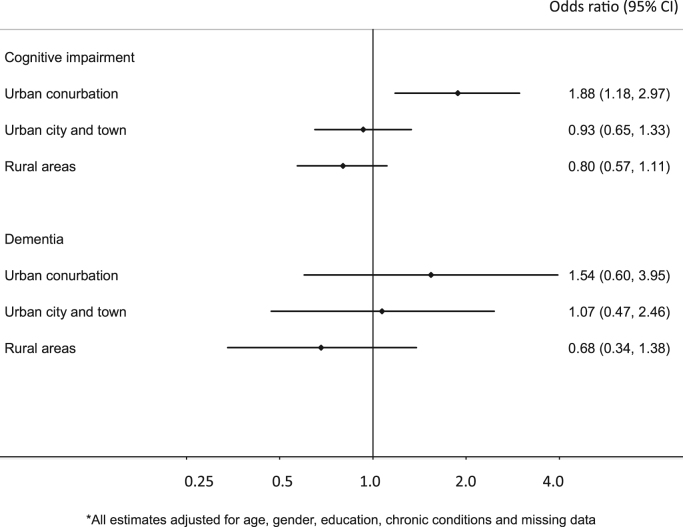
Stratified associations between cognitive disorders and REAT groups (high vs low) across three rural/urban categories.

Table 2 reports the differential relationships between the binary REAT groups (high vs low) and cognitive disorders in the three rural/urban settings (p-value for interaction terms<0.01). In urban conurbations, people living in areas with high REAT scores had 1.88 (95% confidence interval (CI): 1.18, 2.97) times higher odds of cognitive impairment (MMSE≤25) compared to the low REAT group. In urban city and town areas, the odds ratio for cognitive impairment was higher than in urban conurbations but the difference in odds ratio between results with high (odds ratio (OR): 2.27, 95% CI: 1.44, 3.57) and low (OR: 2.11, 95% CI: 1.31, 3.39) REAT score was small. People living in rural areas generally had over twice as higher odds of cognitive impairment than those living in urban conurbations but the direction of association was the opposite. Fig. 1 shows the stratified associations across urban conurbations (1.88; 95% CI: 1.18, 2.97), urban city and town (0.93; 95% CI: 0.65, 1.33) and rural areas (0.80; 95% CI: 0.57, 1.11) (Fig. 1). For dementia, the direction of association was also different across three rural/urban categories although the interaction terms did not achieve statistical significance. Including missing data in the high REAT group did not affect the results.

**Table 2:** The association between cognitive disorders and REAT score.

		Cognitive impairment			Dementia		
		Model 1	Model 2	Model 3	Model 1	Model 2	Model 3
	REAT score	OR (95% CI)	OR (95% CI)	OR (95% CI)	OR (95% CI)	OR (95% CI)	OR (95% CI)
Overall	Low (ref.)	1.00	1.00	1.00	1.00	1.00	1.00
	High	0.96 (0.80, 1.16)	0.94 (0.76, 1.16)	0.95 (0.80, 1.13)	0.87 (0.61, 1.24)	0.90 (0.58, 1.39)	0.78 (0.56, 1.10)
Urban conurbation	Low (ref.)	1.00	1.00	1.00	1.00	1.00	1.00
	High	2.06 (1.37, 3.10)	1.88 (1.18, 2.97)	1.76 (1.18, 2.64)	1.35 (0.62, 2.93)	1.54 (0.60, 3.95)	1.15 (0.53, 2.50)
Urban city and town	Low	2.15 (1.42, 3.27)	2.27 (1.44, 3.57)	2.09 (1.37, 3.19)	1.43 (0.65, 3.11)	1.71 (0.69, 4.23)	1.54 (0.70, 3.39)
	High	2.10 (1.35, 3.24)	2.11 (1.31, 3.39)	2.23 (1.46, 3.40)	1.49 (0.66, 3.40)	1.85 (0.66, 5.17)	1.56 (0.71, 3.45)
Rural areas	Low	2.85 (1.92, 4.23)	3.15 (2.01, 4.91)	2.80 (1.88, 4.17)	1.83 (0.88, 3.79)	2.37 (0.99, 5.72)	1.82 (0.87, 3.84)
	High	2.09 (1.38, 3.16)	2.50 (1.58, 3.98)	2.24 (1.49. 3.36)	1.18 (0.54, 2.62)	1.62 (0.61, 4.31)	1.07 (0.48, 2.37)
p-value for interaction		<0.01	<0.01	<0.01	0.28	0.39	0.26

Model 1: Unadjusted models; Model 2: Adjusted model for age, gender, education, chronic conditions and missing data; Model 3: Missing REAT data in high REAT group; adjusted for age, gender, education and chronic conditions.

The results of depressive and anxiety symptoms are presented in Table 3. The interaction terms between the REAT groups and urban/rural categories did not achieve statistical significance. The associations between common mental disorders and the REAT score did not vary considerably across urban/rural settings. Living in a postcode with high REAT score, with a worse residential environment, was associated with nearly 20% higher odds of depressive (OR: 1.19, 95% CI: 0.99, 1.44) and anxiety symptoms (OR: 1.17, 95% CI: 0.99, 1.38) than living in a postcode with low REAT score. After including missing data in the high REAT group, the overall association between common mental disorders and REAT groups remained similar and achieved statistical significance.

**Table 3:** The association between common mental disorders and REAT score.

		Depressive symptoms			Anxiety symptoms		
		Model 1	Model 2	Model 3	Model 1	Model 2	Model 3
	REAT score	OR (95% CI)	OR (95% CI)	OR (95% CI)	OR (95% CI)	OR (95% CI)	OR (95% CI)
Overall	Low (ref.)	1.00	1.00	1.00	1.00	1.00	1.00
	High	1.18 (1.00, 1.40)	1.19 (0.99, 1.44)	1.21 (1.03, 1.43)	1.17 (1.01, 1.36)	1.17 (0.99, 1.38)	1.18 (1.02, 1.37)
Urban conurbation	Low (ref.)	1.00	1.00	1.00	1.00	1.00	1.00
	High	1.46 (1.06, 2.02)	1.39 (0.98, 1.97)	1.35 (0.98, 1.87)	1.41 (1.05, 1.88)	1.34 (0.98, 1.83)	1.37 (1.03, 1.82)
Urban city and town	Low	0.89 (0.64, 1.25)	0.84 (0.58, 1.22)	0.85 (0.59, 1.20)	0.83 (0.62, 1.13)	0.82 (0.60, 1.12)	0.82 (0.60, 1.11)
	High	1.03 (0.72, 1.46)	1.02 (0.68, 1.51)	0.92 (0.65, 1.31)	0.81 (0.58, 1.11)	0.78 (0.56, 1.10)	0.74 (0.54, 1.02)
Rural areas	Low	0.88 (0.64, 1.21)	0.86 (0.61, 1.21)	0.86 (0.61, 1.19)	0.85 (0.64, 1.13)	0.82 (0.60, 1.10)	0.83 (0.62, 1.11)
	High	0.76 (0.54, 1.07)	0.72 (0.50, 1.05)	0.88 (0.63, 1.24)	0.86 (0.64, 1.16)	0.82 (0.60, 1.13)	0.90 (0.67, 1.21)
p-value for interaction		0.05	0.08	0.43	0.13	0.24	0.13

Model 1: Unadjusted models; Model 2: Adjusted model for age, gender, education, chronic conditions and missing data; Model 3: Missing REAT data included in high REAT group; adjusted for age, gender, education and chronic conditions.

## 4 Discussion

### 4.1 Main findings

This study investigated the associations between the quality of micro-scale environment and mental health using a multicentre study of older people in England. Higher REAT score (poor quality of micro-scale environment) was generally associated with higher odds of mental health problems in older people. Living in rural settings was associated with higher odds of cognitive disorders but the direction of association with the REAT score varied across the three rural/urban categories. For people living in a postcode with a high REAT score, the odds of cognitive impairment was nearly twice higher than their counterparts in urban conurbations but was 20% lower in rural areas. Living in a postcode with a high REAT score was associated with nearly 20% higher odds of depressive and anxiety symptoms, and the relationships did not appear to vary across rural/urban categories. The estimates did not change substantially after including the missing data in the sensitivity analysis.

### 4.2 Strengths and limitations

This study was based on a large population-based cohort of contemporary older people across urban and rural areas in England. A structured interview was used to assess cognitive function and common mental disorders with consistent diagnostic standards across different areas. The assessment targets the postcode areas closest to the residences of participants and this might address the issue of mismatch between geographical boundaries and activity space. The quality of micro-scale environment was assessed objectively using a valid instrument and the method of visual image audits. This avoided the potential same-source bias of using perceived environmental measurements and improved the limitation of small sample size in the previous UK-based studies (Thomas et al., 2007, Weich et al., 2002).

The causal direction between micro-scale environment and mental health could not be fully determined due to the cross-sectional nature of the data. The direction of association might be reversed if people with mental health problems moved to areas with a poor quality environment or have less capacity to maintain their property and local environment. Information on recent relocations was not available in the CFAS II interview. Although the number of people with complete data was over 3000, the statistical power to test the significance of effect sizes might be still limited, particularly for dementia. This analysis did not further include area deprivation or other small area level factors as the two sets of environmental measurements were based on different levels and aspects of the living environment. Since the role of small area level factors in the association was not clear, adding all these environmental factors might be inappropriate and lead to over-adjustment bias (Chaix et al., 2010).

Although REAT is a validated instrument which provides a simple assessment of several micro-scale features, it might not be sufficient to describe the variety of property and street level characteristics. For example, flats are likely to be main type of properties in highly urban areas. The height of flats has been suggested to be related to the concept of defensible space but is not covered by the REAT (Normoyle and Foley, 1988). The visual nature of environmental assessment tool was unlikely to capture some features such as traffic noise, the availability of local support services and opportunities for social participation. Due to limited resources, the environmental assessment was only conducted to a subsample of participants from Cambridgeshire and Nottingham and did not include those from Newcastle upon Tyne, a large metropolitan area in north with different settings. Although the findings of this analysis might not be generalisable to various environmental contexts across different regions and countries, the study areas have covered a wide variety of environmental contexts in central and east England.

### 4.3 Micro-scale environment and common mental disorders

High REAT score was consistently associated with higher odds of common mental disorders across rural/urban settings. The findings provide supportive evidence on the potential influence of social disorder on depressive and anxiety symptoms in later life. Older people living in areas with poor micro-scale features may experience stress, insecurity and lack of control, with a potential impact on mental health and well-being (Julien et al., 2012). Existing reviews on neighbourhood and depression have suggested that the physical characteristics of social disorder may influence individual mental health by disrupting mobility and social support in local areas (Mair et al., 2008, Blair. et al., 2014). Recent studies also reported that older people who perceived there to be a high level of social disorder in their neighbourhood had increased odds of insomnia, poor self-rated health and well-being, which could be related to stress and mental health problems (Chen-Edinboro et al., 2014, Ward Thompson et al., 2012).

Previous studies have reported inconsistent relationships between micro-scale features and depression in the general population after adjusting for individual factors (Weich et al., 2002, Thomas et al., 2007, Araya et al., 2007, Galea et al., 2005). In particular, the investigation including 1058 individuals aged 16–75 in Wales did not find significant associations between REAT scores and symptoms of common mental disorder, which was measured by the 12-item General Health Questionnaire (Thomas et al., 2007). In addition to difference in sample size and measures for mental disorders, various results between previous and current studies might be related to difference in the age of study populations. Compared to younger adults, older people generally spend more time in their local areas and therefore the quality of micro-scale environment might have a stronger impact on mental health in later life (Phillipson, 2012).

### 4.4 Micro-scale environment and cognitive disorders

The association between micro-scale environmental features and cognitive disorders was moderated by rural/urban settings. The differential associations between cognitive disorders and the REAT score might correspond to the potential non-linear relationship for features of land use at the small area level, suggesting both high and low levels of land use mix were associated with increased odds of cognitive impairment (Wu et al., 2015b). Although areas with mixed land uses might be more stimulating environments (Cassarino and Setti, 2015), these features can also be related to environmental stressors in urban areas, such as high level of social disorder. The measurement of the micro-scale environment may capture these elements. In urban conurbations, living in postcodes with high REAT score was associated with higher odds of cognitive impairment and dementia. Poor micro-scale features and a high level of social disorder might be potential environmental stressors which have a negative impact on both emotional and cognitive health. Long-term stress and lack of control might cause repetitive negative thinking and obscure normal cognitive functioning (Marchant and Howard, 2015).

This analysis found that living outside urban conurbations was associated with higher odds of cognitive disorders. These results correspond to some of the evidence in the literature (Cassarino et al., 2016, Nunes et al., 2010, Russ et al., 2012) as well as our previous analysis which showed higher odds of cognitive impairment in areas with low levels of land use mix (Wu et al., 2015b). However, a high REAT score, which indicates poor quality of micro-scale environment, was associated with lower odds of cognitive impairment and dementia. Based on experience of environmental assessment, postcodes with high REAT score were usually on the ‘high streets’ in rural areas. These postcodes had higher property density and were close to local services and resources such as community centres, cafés, shops and public transport. Older rural residents living in this type of postcode might have better access to these basic services, allowing them to have better social interactions in local communities or travel to other areas to visit their friends and family. On the contrary, some environmental characteristics, such as a high level of defensible space and extremely spacious arrangement of houses, are considered to be high quality indicators but may be barriers to social interaction in rural settings, with the consequence of isolation and loneliness, which are important issues in rural ageing and potential risk factors for cognitive decline (UK Government, Department for Environment, Food and Rural Affairs, 2013b; Institute of Medicine, 2015).

### 4.5 Cognitive and common mental disorders: different relationships in rural settings

Micro-scale environmental features seem to have different relationships with cognitive and common mental disorders in rural areas. The reasons for this warrant further investigation on the underlying mechanisms in various types of rural settlements. The interplay of multiple environmental characteristics might provide possible explanations on these differences. For example, although living in rural areas might be related to isolation and loneliness, this analysis and previous studies did not find clear urban/rural differences in the prevalence of depression at older age when taking into account socio-demographic factors (McDougall et al., 2007, Walters et al., 2004). Environmental features, such as green space, in rural areas might have a protective effect on common mental disorders (Astell-Burt et al., 2014, Wu et al., 2015c) and compensate the impact of restricted social interaction on emotional health. The ‘high streets’ in rural areas could be supportive environments with better access to local services and opportunities for social interaction. However, this type of areas might also have heavy traffic and noise, which have been identified to be risk factors for depressive symptoms in older people (Julien et al., 2012, Orban et al., 2016) and might have a stronger impact on emotional than cognitive health in the short term.

### 4.6 Future research directions and public health implications

This study provides evidence on the potential environmental determinants related to cognitive and common mental disorders in older people. Adequate maintenance of streets and properties and removal of the physical characteristics of social disorders might have a positive influence on reducing the risk of mental health problems, particularly in conurbations and metropolitan areas. Public health interventions may incorporate the environmental aspect to improve the quality of local environment as well as support mental health in later life. In rural areas, environmental stimulation might be an important component to maintain cognitive function in later life (Cassarino and Setti, 2015). Local service provision such as public transport, healthcare services and community centres and home maintenance may support daily activity, physical and cognitive functions of older rural residents (UK Government, Department for Environment, Food and Rural Affairs, 2013b).

Potential pathways from environmental factors to individual mental health need to be further investigated in longitudinal studies. To explore mechanisms, it is important to consistently measure the trajectory of physical and mental health status, including robust measurement of lifestyle factors and recording of information on relocation over time. The interaction between older people and their local environments may be different when comparing the younger and older old, and between those with and without disability (Clarke and George, 2005; Phillipson, 2012). These individual characteristics might hence modify the association between micro-scale environment and mental health in later life. Environmental characteristics and the standard for ‘good quality’ of micro-scale environments appear to be different across larger environmental contexts such as urban/rural areas, regions or countries. Future research may incorporate environmental characteristics at micro-scale and small area levels, urban/rural settings as well as wider societal contexts of economic, political and cultural variation in order to provide a comprehensive understanding of environmental influences on mental health in later life.
