# Supplementary material for: Micro-scale environment and mental health in later life: Results from the Cognitive Function and Ageing Study II (CFAS II)
Source: J Affect Disord. 2017 Aug 15;218:359–64. doi: 10.1016/j.jad.2017.05.001 (PMC5478740; doi:10.1016/j.jad.2017.05.001)
Supplement: Supplementary file 1 — Supplementary material [file mmc1.docx]

**Micro-scale environment and mental health in later life: results from the Cognitive Function and Ageing Study II (CFAS II)**

**Supporting information**

**S1. The investigation of postcodes with missing REAT data**

To address the issue of missing data which were found during the data collection of visual image audits, a small amount of these postcodes (N=34) were sampled and assessed through physical audits based on the REAT manual in order to investigate whether the REAT score of these postcodes were different from those with complete data. Table S1 compared the distributions of REAT scores across postcodes with completed and missing data. The results of physical audits in the reliability study (Wu et al. 2014) are also reported in the table. These 43 postcodes had available streetscape images for the REAT assessment. Postcodes with unavailable Google Street View images generally had higher REAT scores compared to those with available images and this difference was unlikely to be caused by different assessment methods.

**Table S1 A comparison of REAT scores across postcodes with complete and missing data**

|  | Complete data | The reliability study  (Wu et al., 2014) | Missing data |
| --- | --- | --- | --- |
| Assessment method | Visual audits | Physical audits | Physical audits |
| Number of postcodes | 1502 | 43 | 34 |
| Median (IQR) | 15.0 (8.0) | 15.0 (10.0) | 18.5 (7.0) |
| Range (min, Max) | (2.5, 41.0) | (4.0, 35.0) | (7.5, 37.0) |

**Reference**

Wu, Y.-T., Nash, P., Barnes, L.E., Minett, T., Matthews, F.E., Jones, A., Brayne, C., 2014. Assessing environmental features related to mental health: a reliability study of visual streetscape images. BMC Public Health 14, 1094.
